# Influence of Temperature Chronobiology on Stroke Outcome

**DOI:** 10.3390/ijms24043746

**Published:** 2023-02-13

**Authors:** Maria Luz Alonso-Alonso, Ana Sampedro-Viana, Manuel Rodríguez-Yáñez, Iria López-Dequidt, José M. Pumar, Antonio J. Mosqueira, Sabela Fernández-Rodicio, Marcos Bazarra-Barreiros, Tomás Sobrino, Francisco Campos, José Castillo, Pablo Hervella, Ramón Iglesias-Rey

**Affiliations:** 1 Neuroimaging and Biotechnology Laboratory (NOBEL), Clinical Neurosciences Research Laboratory (LINC), Health Research Institute of Santiago de Compostela (IDIS), 15706 Santiago de Compostela, Spain; 2 Stroke Unit, Department of Neurology, Hospital Clínico Universitario, 15706 Santiago de Compostela, Spain; 3 Department of Neuroradiology, Hospital Clínico Universitario, Health Research Institute of Santiago de Compostela (IDIS), 15706 Santiago de Compostela, Spain; 4 NeuroAging Group (NEURAL), Clinical Neurosciences Research Laboratory (LINC), Health Research Institute of Santiago de Compostela (IDIS), 15706 Santiago de Compostela, Spain; 5 Translational Stroke Laboratory (TREAT), Clinical Neurosciences Research Laboratory (LINC), Health Research Institute of Santiago de Compostela (IDIS), 15706 Santiago de Compostela, Spain

**Keywords:** circadian rhythm, chronobiology, functional outcome, stroke, temperature

## Abstract

The circadian system regulates numerous physiological variables, including body temperature. Additionally, a circadian patter has been described in stroke onset. Considering this, we hypothesised that the chronobiology of temperature may have an impact on stroke onset and functional outcomes. We also studied the variation of blood biomarkers according to stroke onset time. This is a retrospective observational study. Of the patients included, 2763 had a stroke between midnight and 8:00 h; 1571 between 8:00–14:00 h; and 655 between 14:00 h and midnight. Axillary temperature was measured at admission. At this time, blood samples were collected for biomarker analysis (TNF-α, IL-1β, IL-6, IL-10, and glutamate). Temperature was higher in patients admitted from 8:00 h to midnight (*p* < 0.0001). However, the percentage of poor outcome at 3 months was highest in patients from midnight to 8:00 h (57.7%, *p* < 0.001). The association between temperature and mortality was highest during night time (OR: 2.79; CI 95%: 2.36–3.28; *p* < 0.001). These patients exhibited high glutamate (220.2 ± 140.2 µM), IL-6 (32.8 ± 14.3 pg/mL) and low IL-10 (9.7 ± 14.3 pg/mL) levels. Therefore, temperature chronobiology could have a significant impact on stroke onset and functional outcome. Superficial body hyperthermia during sleep seems to be more dangerous than during wakefulness. Further studies will be necessary to confirm our data.

## 1. Introduction

The circadian system is involved in the regulation of numerous physiological variables, including inflammation, immune response, blood pressure, heart rate, circulating catecholamines, blood coagulation markers, vascular endothelial function and body temperature [1,2,3,4,5]. It is known that during the final hours of sleep, circulating cortisol levels rise and core body temperatures rise, preparing the metabolic and circulatory systems for sudden increases in energy expenditure and activity soon after awakening. These anticipatory alterations imply that physiological responses are not ideal in the absence of the circadian system properly priming or dampening responses based on the time of day or night, possibly as a result of delays in some negative-feedback regulatory systems [6,7]. Current data suggest that circadian disruption has a negative impact on human health [3,4,8,9,10]. Thus, alterations in the circadian rhythmicity of body temperature could have a clinical association with different kinds of diseases and their severity, including trauma, infection, cancer and inflammatory response [11]. In fact, an increase in body temperature has been significantly associated with severity, mortality and poor outcomes in stroke survivors [12,13,14,15,16,17,18,19,20,21].

On the other hand, circadian patter, defined as biological changes that follow a 24 h cycle, has also been widely reported in the onset of stroke, showing higher risk in the morning. This well-established temporal risk was observed for ischemic and haemorrhage strokes [22,23], although some slight differences were observed in stroke TOAST (Trial of Org 10,172 in Acute Stroke Treatment) subtypes [24]. The consideration of circadian rhythms may be necessary in order to optimise therapeutic approaches due to the variability of patient response to ischemic stroke and its therapies as well as factors explaining the lack of consistency with the patients’ evolution [25]. Interestingly, physiological body temperature starts to increase in the morning [11], coinciding with the period when the peak of stroke has been described [22,23,24], and decreases during the sleep period, when the incidence of stroke is lower [26].

Based on these facts, we hypothesised that the chronobiology of body temperature may have an impact on stroke outcome. Therefore, the purposes of this study were to analyse the possible association between body temperature and stroke onset time, and its impact on functional outcome at 3 months. We also studied the variation of blood biomarkers according to stroke onset time.

## 2. Results

### 2.1. Sample Description

**Figure 1:**
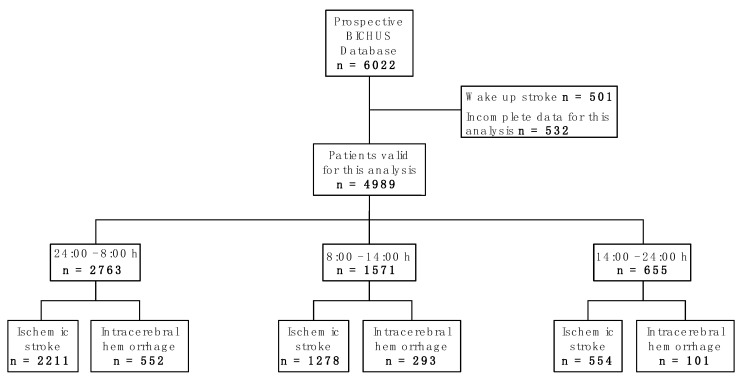
Flowchart of patient screening.

**Figure 2:**
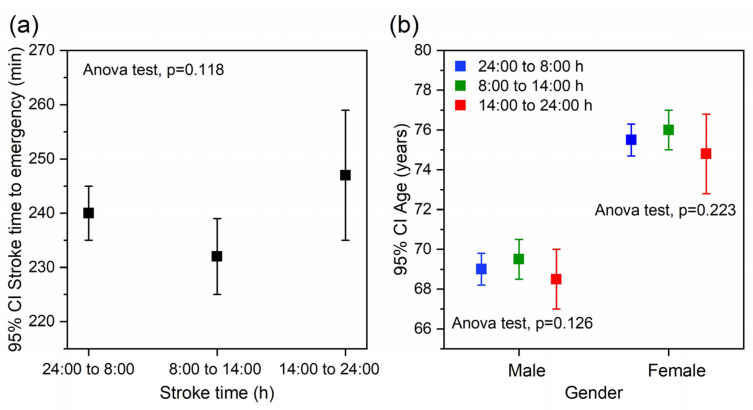
Relationship between stroke time and (**a**) time to emergency care and (**b**) age with respect to gender.

The Biobanco Ictus del Complejo Hospitalario Universitario de Santiago (BICHUS) data bank includes a total of 6022 patients with ischemic stroke and intracerebral haemorrhage. For the present study, we excluded wake-up stroke patients and those individuals whose register did not include the necessary data for this analysis (*n* = 1033). Of the 4989 patients finally included, 2763 had suffered stroke between midnight and 8:00 h, 1571 between 8:00 h and 14:00 h, and 655 between 14:00 h and midnight (Figure 1). The time elapsed between the stroke and emergency care tended to be higher from 14:00 h to 8:00 h than from 8:00 h to 14:00 h. However, the differences were not significant (Figure 2a). There were also no significant differences in age when comparing the selected times for each gender. Nevertheless, the age of the patients who experienced a stroke between 14:00 h and midnight tended to be lower for both genders (Figure 2b).

**Figure 3:**
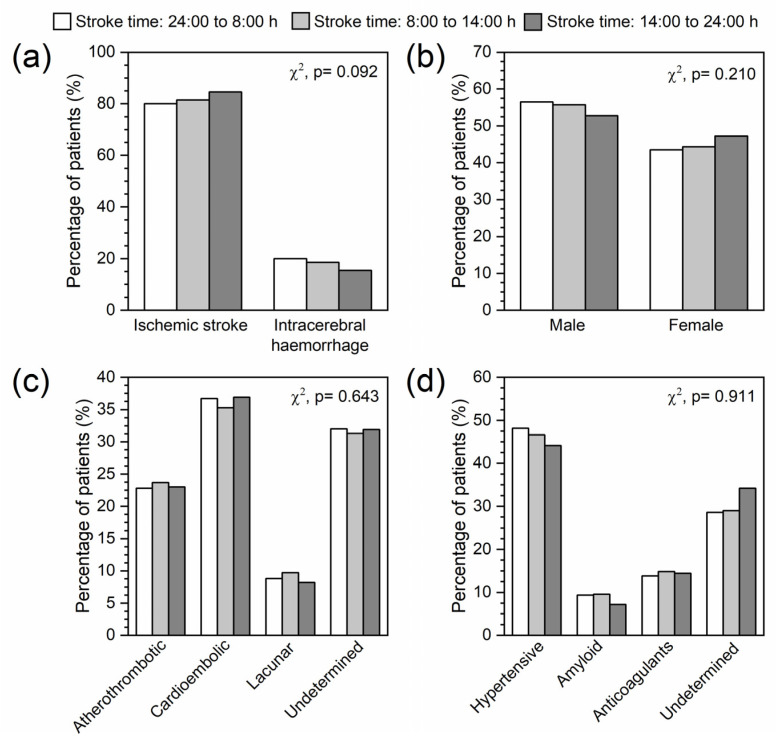
Relationship between stroke time and (**a**) type of stroke, (**b**) gender and etiology of (**c**) ischemic stroke and (**d**) intracerebral haemorrhage.

Although the percentage of patients with ischemic stroke tended to increase throughout the day, and those with intracerebral haemorrhage tended to decrease (Figure 3a), our results show that timing has no significant impact on the type of stroke. Likewise, the distribution of gender is similar regardless of the stroke time (Figure 3b), tending to be higher early in the day in men and at the end of the day in women. There were also no significant differences in the etiology of either ischemic stroke or intracerebral haemorrhage in relation to the time of stroke (Figure 3c,d).

### 2.2. Association between Temperature, Stroke Time, and the Functional Outcome at 3 Months

**Figure 4:**
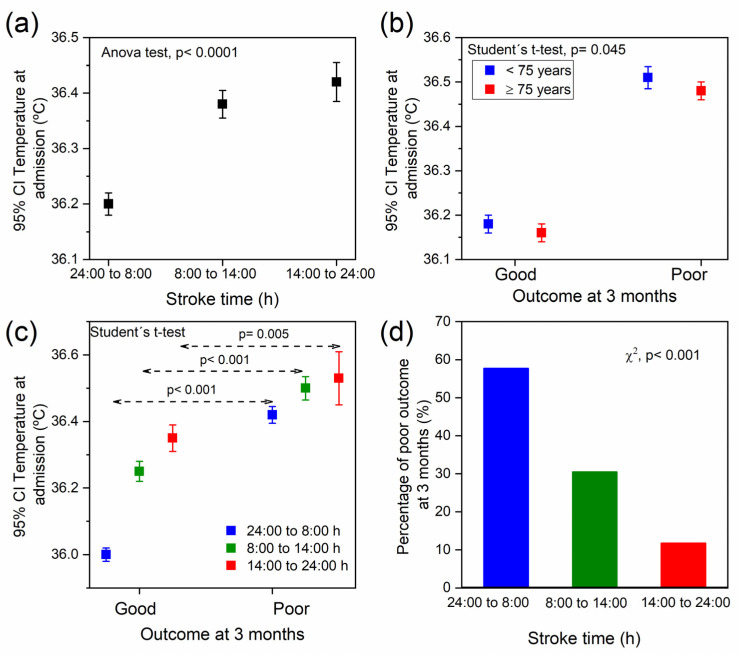
Relationship between temperature at admission and (**a**) stroke time, (**b**) outcome at 3 months with respect to age. (**c**) Relationship between temperature at admission and outcome at 3 months and (**d**) percentage of poor outcome at 3 months both with respect to stroke time.

The results show that the body temperature was increased throughout the day, being significantly higher in the patients admitted from 8:00 h to midnight than those admitted from midnight to 8:00 h (Figure 4a; *p* < 0.0001). On the other hand, it has been shown that higher temperature at admission was related with poor outcome at 3 months regardless of the patient’s age (Figure 4b). When this relationship was analysed taking into account the period of time in which the stroke occurred, it was observed that higher temperature was significantly associated with poor outcomes at 3 months in all stroke times studied. However, the temperature difference between those patients with good and poor outcomes at 3 months was higher in those admitted from midnight to 8:00 h than those admitted from 8:00 h to midnight (Figure 4c). Contrary to what may be expected from these data, the total percentage of patients with poor outcomes at 3 months was significantly higher in those who suffered the stroke from midnight to 8:00 h (57.7%; *p* < 0.001). This percentage was reduced to 30.5% from 8 to 14:00 h, and to 11.8% from 14:00 h to midnight (Figure 4d).

**Figure 5:**
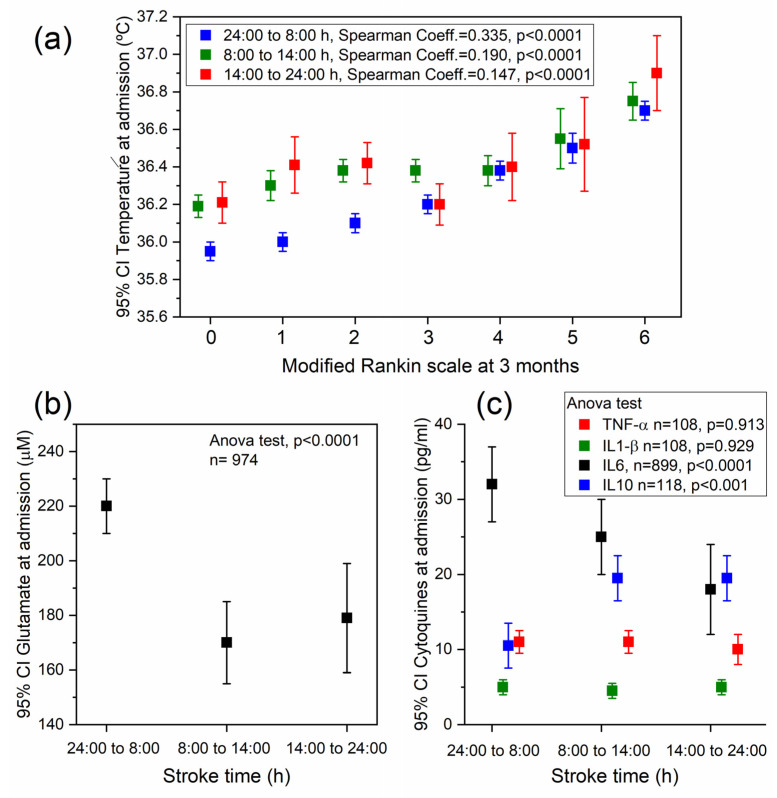
(**a**) Relationship between temperature at admission and modified Rankin scale at 3 months. Relationship between stroke time and (**b**) plasma glutamate and (**c**) cytokines at admission.

The previous data were supported by the significant correlation shown between temperature at admission and the modified Rankin scale (mRS) for all stroke times (Figure 5a). The Spearman’s rank coefficients were 0.335 for stroke time from midnight to 8:00 h, 0.190 from 8:00 h to 14:00 h, and 0.147 from 14:00 h to midnight. Therefore, although the temperature of patients admitted during the first 8:00 h was lower, the association between temperature at admission and mRs was stronger than in strokes occurring during the rest of the day.

Moreover, the results of the logistic regression model for temperature and NIHSS at admission, age, and reperfusion treatment show a significant association between all variables analysed and poor outcome at 3 months (*p* < 0.001) except for temperature at admission in those patients who suffered a stroke between 14:00 h and 24:00 h (Table 1). Thus, these data showed that the association between the temperature at admission and higher rate of mortality increment was stronger with stroke time from midnight to 8:00 h (OR: 2.79; CI 95%: 2.36–3.28; *p* < 0.001) than from 8:00 h to 14:00 h (OR: 1.52; CI 95%: 1.26–1.85; *p* < 0.001), and from 8:00 h to midnight (OR: 1.23; CI 95%: 0.94–1.60; *p* = 0.132).

### 2.3. Association between Stroke Time and Different Biomarkers

The analysed molecular markers indicated that patients who suffered a stroke from midnight to 8:00 h had higher level of glutamate (24:00/8:00 h: 220.2 ± 140.2 µM, 8:00/14:00 h: 167.2 ± 139.6 µM and 14:00/24:00 h: 177.6 ± 147.3 µM; Figure 5b, *p* < 0.001), IL-6 (24:00/8:00 h: 32.8 ± 14.3 pg/mL, 8:00/14:00 h: 19.1 ± 12.8 pg/mL and 14:00/24:00 h: 21.3 ± 9.0 pg/mL Figure 5c, *p* < 0.0001) and lower levels of IL-10 (24:00/8:00 h: 9.7 ± 14.3 pg/mL, 8:00/14:00 h: 19.1 ± 12.8 pg/mL and 14:00/24:00 h: 21.3 ± 9.0 pg/mL; Figure 5c, *p* = 0.001). Regarding TNF-α and IL1-β, there were no level differences related to the stroke time.

## 3. Discussion

In the present study, we evaluated the chronology of the temperature in stroke patients. It was analysed whether there was a difference in temperature among patients according to the time of admission. It has been shown that both the stroke and body temperature have a circadian rhythm [2,27]. Temperature has a significant impact on the clinical course of stroke patients. Thus, higher temperature leads to poor outcomes in stroke [12,13,14,15,16,17,18,19,20,21]. In addition, the increase in body temperature could affect the efficacy of reperfusion in these patients [17,28]. Our results show that the influence of body temperature on stroke outcome seems to have a chronobiological pattern.

The disruption of the temperature circadian rhythm has been associated with different diseases and their severity [4,10,11]. The alteration of this circadian rhythm is observed frequently in elderly persons [29,30,31]. This population has more difficulties to decrease the cerebral temperature during overnight [32]. Body temperate is also elevated in the elderly who suffer from being poor sleepers [33]. In fact, sleep disorders has been related to both ischemic and haemorrhagic stroke [34]. In this sense, our results show that temperature at admission was similar in patients under and over 75 years, although this was higher in those with poor outcome at 3 months. However, the sample analysed was composed of patients of fairly advanced age. In fact, in the decade in which the patients were admitted, a significant increase in the age of women with stroke was observed in this part of Spain [35].

Our results confirm that age was higher in women regardless of stroke time. However, the percentage of men was slightly higher at all time points. No differences were observed in elapsed time from stroke event to emergency admission according to stroke time. Thus, although the increase in this period is a crucial factor for the clinical outcomes [36,37], this factor was not a variable that may lead to the misinterpretation of our results.

Regarding the daily distribution of stroke events, there was a slight tendency to increase ischemic stroke throughout the day. The opposite tendency was observed in the cases of intracerebral haemorrhage. When the daily distribution of both types was analysed according to their etiology, no significant differences were observed. This supports the data previously described by Jimenez-Conde et al. in 2017 [26]. However, Ripamonti et al. [24] had shown that all ischemic stroke subtypes were more frequent in the morning, except cardioembolic stroke, which was less numerous in the late morning.

The temperature was higher in patients admitted from 8:00 h to midnight. This is coherent with the daily circadian rhythm of body temperature, in which the highest temperature is reached around 18 h and the lowest around 6 h [11]. Interestingly, the peak of ischemic stroke risk was stablished between 6 and 12 h [22], in the time when the temperature is still low, but begins to rise [11]. On the other hand, higher body temperature has been widely associated with poor outcomes [12,13,14,15,16,17,18,19,20,21], which was also observed in our results regardless of the stroke time. However, our data also showed that the percentage of poor outcomes at 3 months was significantly higher precisely in the group of patients admitted at night, which had exhibited the lowest temperature at admission. Moreover, a stronger correlation was observed between mRs and poor outcome at 3 months with the temperature increase in this day period than in the rest of the day. It seems that body hyperthermia during sleep is more dangerous than during wakefulness.

Recently, the association between the time of day and the outcome of ischemic stroke patients treated with endovascular thrombectomey has been described [38]. Opposite of our results, in these patients, good outcomes at 3 months were observed in those who suffered ischemic stroke at night. This study proposed that the lower body temperature at night, among other factors, may have had a potential impact on their results [38]. Conversely, other studies have claimed that, although suffering a stroke at night is less frequent, they usually are more severe [26,39] and exhibit faster infarct progression [40], which supports the data observed in the present study.

Finally, there is an increasing interest in the identification of stroke blood biomarkers, which could help in diagnosis, prognosis and management of these patients [41,42,43,44]. In this study were analysed glutamate, IL-1β, IL-6, IL-10 and TNF-α according to the time of stroke. The level of glutamate and IL-6 were elevated in patients with stroke onset between midnight and 8:00 h. The level of IL-10 was lower in this group. These changes in such biomarkers had been previously related with bad prognosis in stroke [45,46,47,48,49,50,51], which supports the poor outcomes observed in our sample of patients. On the other hand, the circadian rhythm could also be involved in the 24 h level variation of these biomolecules. Previously, it has been shown that IL-6 exhibited the highest levels at night [52,53,54], which supports our observations. Similarly, higher levels of glutamate have been detected in evenings than in mornings [55,56]. By contrast, the levels IL-10 seems to be lowest at night [53]. Thus, these endogenous variations of IL-6, IL-10 and glutamate could play an important role in the outcome of these patients. Regarding IL-1β and TNF-α, although it has been claimed that they release more at night [53,54], this difference was not detected in our patients.

As far as we know, this is the first study that shows that the influence of body temperature on stroke outcome has a circadian rhythm. Temperature is an easily measured parameter in general clinical practice, and therefore, considering the chronobiology of this factor may be a tool to approach and treat stroke patients.

The main limitations of this study are related to its retrospective design and to the fact that it is a single-centre study. In this work, we study the relationship between stroke time with superficial body temperature, but not with brain temperature. Although cerebral temperature also seems to be increased in stroke patients, and therefore it could have an effect on stroke outcomes [57], this is not a parameter routinely measured in the clinical examination of these patients. Additionally, the variable of available human resources, which are generally lower on night shifts, and the season in which the stroke was onset, were not included in the analysis. The weight variable was also not included in the analysis, although a previous study has shown its relationship with temperature [58]. The strengths of this work are the unbiased selection of patients, the high inclusion rate following selection criteria and the blinded analysis of data.

## 4. Materials and Methods

### 4.1. Study Design

This is a retrospective observational analysis of patients with ischemic stroke admitted to the Stroke Unit of the Hospital Clínico Universitario of Santiago de Compostela (Spain), who were prospectively registered in an approved data bank, BICHUS. All patients were treated by expert neurologists according to national and international guidelines. An accredited neurologist (MR-Y, IL-D) assessed the clinical scales of the National Institute of Health Stroke (NIHSS) upon admission and the mRS [59] at hospital discharge and at 3 months. We consider a poor outcome a mRS ≥ 2 in the evaluation at 3 months. Stroke diagnosis was made using the TOAST criteria [60].

The main objective of this study was to analyse the association between body temperature and stroke onset time evaluated by its impact on functional outcome at 3 months. Secondary endpoints were the association between stroke onset time and serum levels of tumour necrosis factor alpha (TNF-α), interleukin 1 beta, (IL-1β), IL-6, IL-10 and excitotoxicity (glutamate).

### 4.2. Inclusion and Exclusion Criteria

Inclusion criteria for this analysis were: (1) authorisation for the anonymous use of individuals’ data for research purposes, (2) temperature control and (3) follow-up at 3 months. We excluded wake-up stroke patients and those individuals whose register did not include the necessary data for this analysis.

### 4.3. Standard Protocol Approvals, Registrations and Patients Consents

This study was carried out in accordance with the Declaration of Helsinki of the World Medical Association (2008) and approved by the Ethics Committee of Santiago de Compostela: (2019/616). Data analysis for this study was retrospective and ran from January 2008 to December 2017. Written informed consent from each patient, or from their relatives, was obtained prior to the start of the study.

### 4.4. Temperature Control and Biochemical Analysis

For this analysis, the patient’s axillary temperature was measured by the nursing staff at admission to the Stroke Unit. The temperature at admission to the stroke unit was selected for this analysis.

TNF-α, IL-1β, IL-6, IL-10 and glutamate biomarkers were performed in the Clinical Neurosciences Research Laboratory by researchers blinded to clinical data. Blood samples, obtained from all patients at admission, were collected in test tubes, centrifuged at 3000× *g* during 15 min and immediately frozen/stored (at −80°C). Serum concentrations of IL-6 were determined by enzyme-linked immunosorbent assay (ELISA) technique following manufacturer’s instructions (BioLegend, San Diego, CA, USA), minimum assay sensitivity 1.6 pg/mL with an intra- and inter-assay coefficient of variation (5.0% vs. 6.8%). Serum levels of IL-10 were quantified using the enzyme-linked immunosorbent assay technique following manufacturer’s instructions (BioLegend, San Diego, CA, USA). TNFα and IL-1β were measured using an immunodiagnostic IMMULITE 1000 System (Siemens Healthcare Global, Los Angeles, CA, USA). Minimum assay sensitivity was 1.7 pg/mL, with an inter-assay CV of 6.5% and intra-assay CV of 3.5%. Serum glutamate concentration was determined by high-performance liquid chromatography (1260 Infinity II, Agilent Technologies, Santa Clara, CA, USA) using the AccQ-Tag™ Precolumn derivatisation method for amino acid analysis (Waters, Milford, MA, USA), following a previously described method [61].

### 4.5. Statistical Analyses

For the descriptive study of the quantitative variables, we used the mean ± one standard deviation or the median [range] according to the type of distribution determined by the Kolmogorov–Smirnov test for a sample with the significance correction of Lilliefors. The significance of the differences was estimated using the student’s *t*-test or the Mann–Whitney U test. One-sided analysis of variance (ANOVA) was used to compare differences between more than two groups. In this line, relationships between stroke time and plasma glutamate and cytokines at admission were evaluated. The qualitative variables were expressed as percentages and for the differences the chi-squared test and, if applicable, the uncertainty coefficient were used.

Correlations between temperature at admission and mRs were performed using Spearman’s rank coefficient. Logistic regression analyses were performed to identify those variables (temperature and NIHSS at admission, age and reperfusion) independently associated with poor outcome at 3 months for different stroke onset times. The results are expressed as odds ratio (OR) with 95% confident intervals (95% CI). A *p* value < 0.05 was considered statistically significant in all analyses. All statistical analyses were performed with SPSS V.21.0 (IBM, New York, NY, USA).

## 5. Conclusions

The results observed in this study allow us to conclude that the body temperature chronobiology could have a significant impact not only on stroke time incidence, but also on the functional outcomes at 3 months. Thus, although the higher risk of the onset of stroke is in the morning, superficial body hyperthermia during sleep seems to be more dangerous than during wakefulness. To our knowledge, this is the first time that the influence of body temperature on all stroke outcomes has been shown to have a circadian rhythm. These results support the necessity of taking into consideration the chronobiology of the body temperature in the approach and treatment of stroke patients. However, further studies will be necessary to confirm our data.

## Figures and Tables

**Table 1:** Logistic regression model for temperature and NIHSS at admission, age, and reperfusion treatment. Dependent variable: poor outcome at 3 months.

**Stroke time from 24:00 to 8:00 h**						
	**Not Adjusted**			**Adjusted**		
	**OR**	**CI 95%**	***p***	**OR**	**CI 95%**	***p***
Temperature at admission	2.76	2.45–3.12	<0.0001	2.79	2.36–3.28	<0.0001
Age	1.04	1.03–1.05	<0.0001	1.03	1.02–1.04	<0.0001
Reperfusion treatment	0.47	0.39–0.56	<0.0001	0.19	0.15–0.24	<0.0001
NIHSS at admission	1.13	1.12–1.15	<0.0001	1.14	1.12–1.16	<0.0001
**Stroke time from 8:00 to 14:00 h**						
	**Not adjusted**			**Adjusted**		
	**OR**	**CI 95%**	***p***	**OR**	**CI 95%**	***p***
Temperature at admission	1.66	1.43–1.91	<0.0001	1.52	1.26–1.85	<0.0001
Age	1.04	1.03–1.05	<0.0001	1.03	1.02–1.04	<0.0001
Reperfusion treatment	0.40	0.31–0.52	<0.0001	0.16	0.12–0.22	<0.0001
NIHSS at admission	1.13	1.11–1.15	<0.0001	1.15	1.13–1.17	<0.0001
**Stroke time from 14:00 to 24:00 h**						
	**Not adjusted**			**Adjusted**		
	**OR**	**CI 95%**	***p***	**OR**	**CI 95%**	***p***
Temperature at admission	1.31	1.08–1.59	0.006	1.23	0.94–1.60	0.132
Age	1.05	1.04–1.07	<0.0001	1.05	1.03–1.06	<0.0001
Reperfusion treatment	0.40	0.22–0.50	<0.0001	0.17	0.10–0.28	<0.0001
NIHSS at admission	1.10	1.07–1.12	<0.0001	0.10	1.06–1.13	<0.0001

## Data Availability

The statistical analysis plan is available upon request. The data bank is not available for legal and ethical reasons.
